# A Novel, Dual-Initiator, Continuous-Suspension Grafting Strategy for the Preparation of PP-g-AA-MAH Fibers to Remove of Indigo from Wastewater

**DOI:** 10.3390/polym16152144

**Published:** 2024-07-28

**Authors:** Sijia Xie, Ziyang Fang, Zhouyang Lian, Zhengwei Luo, Xueying Zhang, Shengxiu Ma

**Affiliations:** 1 School of Environmental Science and Engineering, Nanjing Tech Univeristy, Nanjing 211816, China; 2 Karamay Zhiyuan Bochuang Environmental Protection Technology Co., Ltd., Karamay 834000, China

**Keywords:** suspension grafting, dual initiators, PP-based functional fiber, adsorption, indigo wastewater

## Abstract

The indigo dye found in wastewater from printing and dyeing processes is potentially carcinogenic, teratogenic, and mutagenic, making it a serious threat to the health of animals, plants, and humans. Motivated by the growing need to remove indigo from wastewater, this study prepared novel fiber absorbents using melt-blow polypropylene (PP) melt as a matrix, as well as acrylic acid (AA) and maleic anhydride (MAH) as functional monomers. The modification conditions were studied to optimize the double-initiation, continuous-suspension grafting process, and then functional fibers were prepared by melt-blown spinning the modified PP. The results showed that the optimum modification conditions were as follows: a 3.5 wt% interfacial agent, 8 mg/L of dispersant, 80% monomer content, a 0.8 mass ratio of AA to MAH, a 1000 r/min stir speed, 3.5 wt% initiator DBPH grafting at 130 °C for 3 h, and 1 wt% initiator BPO grafting at 90 °C for 2 h. The highest grafting rate of the PP-g-AA-MAH was 31.2%, and the infrared spectrum and nuclear magnetic resonance spectroscopic analysis showed that AA and MAH were successfully grafted onto PP fiber. This modification strategy also made the fibers more hydrophilic. The adsorption capacity of the PP-g-AA-MAH fibers was highly dependent on pH, and the highest indigo adsorption capacity was 110.43 mg/g at pH 7. The fiber adsorption capacity for indigo increased rapidly before plateauing with increasing time or indigo concentration, and the experimental data were well described in a pseudo-second-order kinetic model and a Langmuir isothermal adsorption model. Most impressively, the modified fiber adsorption capacity for indigo remained as high as 91.22 mg/g after eight regeneration and reuse cycles. In summary, the PP-g-AA-MAH fibers, with excellent adsorption-desorption characteristics, could be readily regenerated and reused, and they are a promising material for the removal of indigo from wastewater.

## 1. Introduction

The textile industry produces large quantities of dyeing wastewater and is a major source of environmental pollution [1]. Compared with other wastewater, printing and dyeing wastewater has the characteristics of difficult biodegradable and complex composition. Generally, the pH of printing and dyeing wastewater is 6–10, ${\mathrm{COD}}_{\mathrm{Cr}}$ is 400–1000 mg/L, ${\mathrm{BOD}}_{5}$ is 100–400 mg/L, SS is 100–200 mg/L, and the chromaticity is 100–400 times, and it always contains a variety of dyes, which is difficult to treat [2,3]. Among dyes, indigo is widely used and has also been identified as a potential carcinogen, teratogen, and mutagen [4,5,6]. The enrichment of indigo in wastewater from the textile industry, therefore, poses a serious threat to the health of animals, plants, and human beings. Therefore, effective treatment strategies that can remove indigo from textile and printing waste are urgently needed.

At present, wastewater containing indigo is treated via coagulation precipitation [7] and electrochemical [8], biochemical [9,10], membrane separation [11], or adsorption methods [12,13,14]. Physical methods, such as adsorption, recycle dyes from wastewater without causing secondary pollution [15,16], making these strategies especially attractive. In particular, adsorption strategies are the treatment method of choice in sudden environmental accidents [17].

While polypropylene (PP) is the most common synthetic material produced on industrial scales with excellent chemical and physical properties [18,19], it is non-polar and, therefore, cannot be used in water treatment applications. To be able to extend the use of PP to this application area, polar functional groups must be added to the PP to make it more polar and more hydrophilic [20,21]. Stanislav Voronov [22] proposed the theory of using a free-radical mechanism to covalently graft polymers in order to modify polyolefins. Currently, most studies use graft polymerization to modify PP [23]. Among grafting polymerization methods, suspension grafting has the advantages of requiring low reaction temperatures, it uses an easily degraded matrix, it can be well controlled, and it can be used with simple post-treatment with water as suspension medium products [24], and as result, it has attracted more research attention in recent years.

Combining the advantages of the aforementioned materials and methods, novel PP-based materials were developed to remove indigo from wastewater in this study. Melt-blown PP resin was used as a matrix, as it is cheap and readily available, and acrylic acid (AA) and maleic anhydride (MAH) were used as functional monomers. The modification conditions were optimized to improve the suspension grafting rate with dual initiators containing 2,5-dimethyl-2,5-dihexane (DBPH) and benzoyl peroxide (BPO). The effects of the amount of initiator, interfacial agent, dispersant, monomer, reaction time, and temperature, as well as other process conditions, on the grafting reaction were investigated. The modified PP was then used to prepare fibers via melt-blown spinning and used to treat wastewater. The adsorption behavior of the fibers was also investigated.

## 2. Materials and Methods

### 2.1. Materials

PP melt-blown resin was purchased from Shanghai Expert in the Developing of New Material Co., Ltd. (Shanghai, China). AA, MAH, DBPH, BPO, anhydrous ethanol, sodium hydroxide (NaOH), hydrochloric acid (HCl), isopropyl alcohol, acetone, and all other reagents were obtained from commercial sources and were of analytical grade.

### 2.2. Preparation of PP-g-AA-MAH Fibers

A predetermined amount of PP resin and xylene, the interfacial agent, was added to a 10-L magnetic-driven reactor. The temperature was then raised to 140 °C to fully dissolve the PP resin in xylene. Subsequently, a predetermined amount of tap water was quickly pumped into the reactor to quench the reaction mixture and disperse the PP particles, and the desired amounts of AA and MAH monomers were added. The reactor was then sealed, it was heated to 130 °C, and the mixture was incubated for 60 min to ensure that the PP swelled. After the PP had swollen, the desired amount of DBPH initiator was added through the pressurized feeding port. After the desired time, the reaction was cooled to a set temperature, and the desired amount of BPO was added to continue the grafting reaction. The reaction products were then recovered via centrifugation and dried. The prepared PP-g-AA-MAH was then used to prepare fibers via melt-blown spinning.

### 2.3. Determination of Product Grafting Ratio and Monomer Grafting Efficiency

The PP-g-AA-MAH fibers were rinsed with pure water and then placed in a Soxhlet extractor and extracted with acetone for 16 h. After drying, 0.5 g of the fibers were removed and placed in a 250-mL round-bottomed flask. Xylene (100 mL) and a KOH/ethanol solution (70 mL) were then added to the round-bottom flask, the mixture was heated until it boiled, and the mixture was refluxed for 30 min. After the reaction, the mixture was cooled to room temperature, and then 2–3 drops of thymol blue indicator were added. The mixture was titrated with an HCl/isopropyl alcohol solution (0.05 mol/L), and the endpoint was defined as the drop-back volume at which the mixture turned egg-yellow. The un-grafted PP was used as the control. The grafting rate (Gr, %) and monomer grafting efficiency (Ge, %) were calculated according to Equations (1) and (2), respectively,

(1) $$Gr=\frac{\left(V_{0}-V_{1}\right)\times c\times \left(98.06+72.06\right)}{3\times m}\times 100\%$$

(2) $$Ge=\frac{m_{AA}^{\prime }+m_{MAH}^{\prime }}{m_{AA}+m_{MAH}}\times 100\%$$

where $V_{0}$ (L) and $V_{1}$ (L) are the volumes of HCl/isopropyl alcohol solution consumed in the titration of the blank and sample, respectively, c (mol/L) is the concentration of the HCl/isopropyl alcohol solution, *m* (g) is the sample mass, $m_{AA}+m_{MAH}$ (g) is the amount of AA and MAH added to the grafting reaction, $m^{\prime }AA+m^{\prime }MAH$ (g) is the content of AA, and MAH in the product.

### 2.4. Characterization Methods

The functional groups present in the PP and PP-g-AA-MAH fibers were identified using Fourier-transform infrared spectroscopy (FT-IR) (Nicolet IS 10). FT-IR data were collected over a wavelength range of 400∼4000 cm^−1^. The micromorphology of PP and PP-g-AA-MAH fibers was observed via scanning electron microscopy (SU-8010, Hitachi (China) Ltd., Beijing, China). Proton nuclear magnetic resonance spectroscopy (^1^H-NMR) data were also collected on the PP and PP-g-AA-MAH fibers using an AVANCE500 instrument. The wetting angle of the materials was measured via the capillary micropressure method and calculated using the Washburn equation [25]. The test device and contact angle correction are described in more detail in previous literature [26,27].

### 2.5. Adsorption and Regeneration of Indigo Using the PP-g-AA-MAH Fibers

The adsorption of indigo using the PP-g-AA-MAH fibers was investigated using static adsorption experiments. For the adsorption experiments, simulated indigo wastewater was placed in tapered bottles with plugs. A defined mass of PP-g-AA-MAH fiber was added to the flask, and the flask was sealed with plastic wrap and placed in a 25 °C constant-temperature oscillator. The fibers were removed from the solution at predetermined time points, and the concentration of indigo in the solution was measured at 610 nm in a UV-visible spectrophotometer [23]. The adsorption capacity (Q, mg/g) was calculated using Equation (3).

(3) $$Q=\frac{(C_{0}-C_{1})\times V}{m}$$

where $C_{0}$ (mg/L) and $C_{1}$ (mg/L) are the concentration of indigo in the solution before and after the adsorption of the PP-g-AA-MAH fibers, *V* (L) is the volume of the solution, and *m* (g) is the mass of the fiber as an absorbent.

To study the desorption of the indigo dye from the PP-g-AA-MAH fibers, fibers saturated with dye were placed in an HCl (0.5 mol/L) solution and shaken for a predetermined amount of time. The indigo concentration in the solution was then determined, and the desorption rate (D, %) was calculated according to Equation (4).

(4) $$D=\frac{V_{2}\times C_{2}}{m\times e_{e}}\times 100\%$$

where $C_{2}$ (mg/L) is the concentration of indigo in the eluent, $V_{2}$ (L) is the volume of the eluent, *m* (g) is the mass of the fiber, and $q_{e}$ (mg/g) is the adsorption capacity. All the experiments were performed in triplicate, and the presented results are the average values.

## 3. Results and Discussion

### 3.1. Mechanism of Suspension Grafting Polymerization

**Figure 1:**
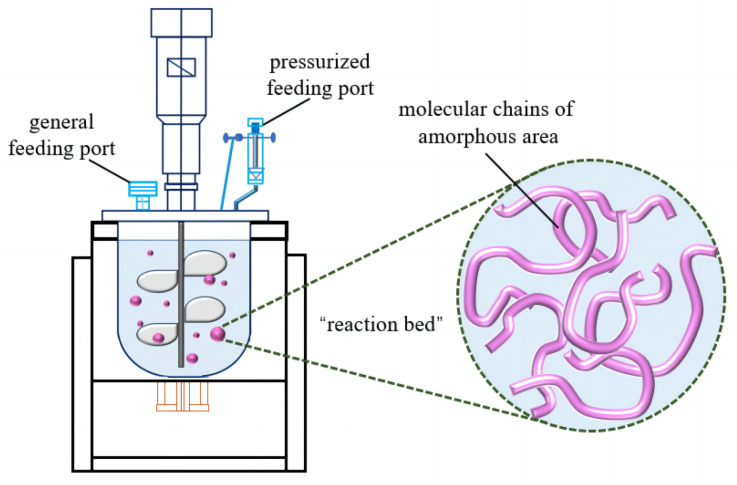
Illustration of the suspension-grafting reaction system.

Figure 1 shows a schematic illustration of the suspension grafting polymerization reaction. Xylene and PP resin were added to a 10-L reactor, and the suspension was magnetically stirred. The mixture was heated until the PP dissolved, and then water was pumped into the reactor to quickly cool the PP. The quenching pretreatment step was performed to increase the amount of amorphous PP in the sample, as well as to increase the degree of swelling, and therefore the surface area of the sample, for the grafting polymerization reaction because the small-molecule initiators and monomers could more easily diffuse into voids on the surfaces of the swollen PP. Then, the PP particles, xylene, water, and grafted monomer were fully mixed at high stir speeds to form many “reaction bed” sites. The suspension–polymerization grafting reaction primarily occurred in the amorphous regions of the PP particles.

**Figure 2:**
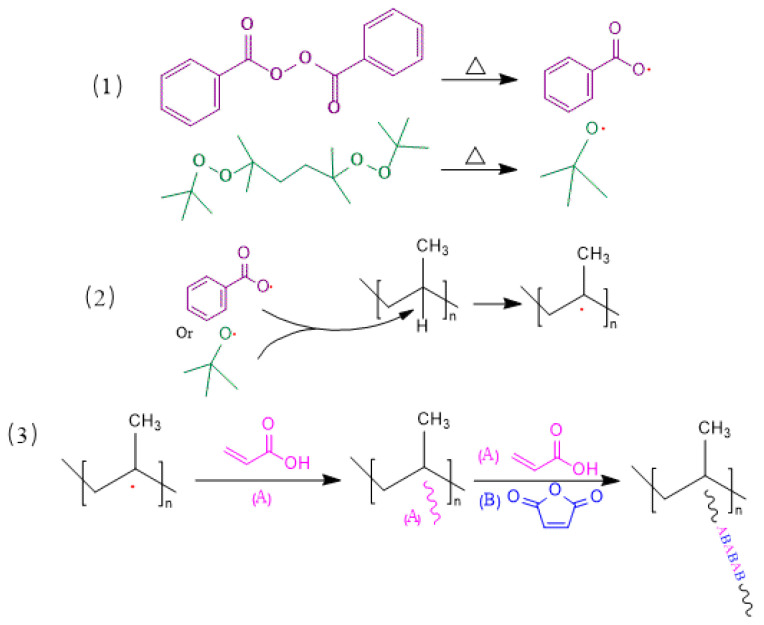
Suspension-grafting polymerization mechanism of AA and MAH monomers.

The mechanism of the suspension-grafting polymerization reaction is shown in Figure 2. First, the initiator was decomposed into primary free radicals at high temperatures (reaction 1). These free radicals then grabbed α-H on the PP backbone to form a PP free radical (reaction 2). The PP free radical then reacted with monomers to initiate the grafting polymerization reaction (reaction 3). With strong reactivity and providing electrons for a reaction system, AA monomers were the first to react with the PP free radicals, and the grafted AA groups increased the intermolecular interactions between the polymer chains, thereby preventing the degradation of the PP and minimizing other side reactions. The stably grafted AA groups then reacted with other AA or MAH monomers as the reaction proceeded.

### 3.2. Influence of Reagent Concentrations on the Grafting Reaction

**Figure 3:**
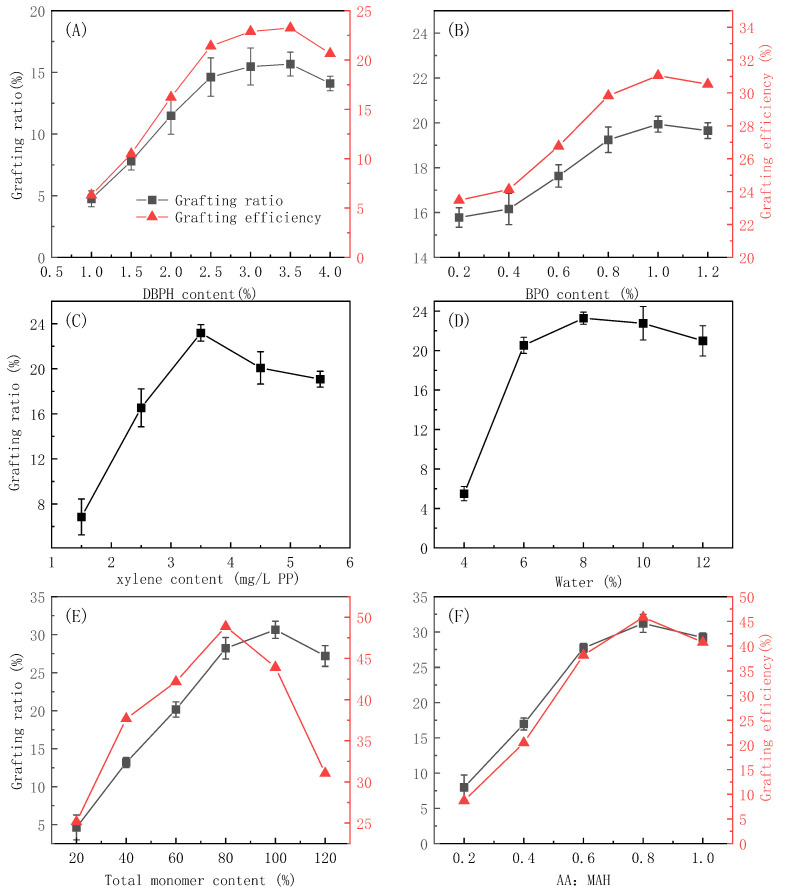
The effects of DBPH (**A**), BPO (**B**), xylene (**C**), water (**D**), the monomer amount relative to PP (**E**), and the ratio of AA to MAH monomers (**F**) on the grafting effectiveness.

Reagent concentrations significantly influence the effectiveness of suspension-grafting polymerization. In this study, the relative amounts of the initiator, interfacial treatment agent, and dispersant, the total dosage of the monomer, and the monomer ratio were investigated as the main influencing factors. The results of these studies are shown in Figure 3.

It can be seen from Figure 3A,B that the concentration of both the DBPH and BPO initiators significantly influenced the grafting reaction. As the amount of initiator increased, the grafting ratio first increased rapidly and then increased more gradually until reaching a maximum value and then decreasing. The same overall trends were also seen in the monomer-grafting efficiency. Initially, increasing the initiator concentration increased the number of free radicals that formed at the reaction temperature, which in turn initiated the polymerization of more monomers and increased the grafting efficiency. However, as the concentration increased further, the concentration of free radicals was too high, and side reactions such as homopolymerization occurred [28,29]. The homopolymerization side reaction consumed a large amount of the monomer, resulting in less monomer grafted to the PP branch chain, which resulted in a decrease in the grafting rate and grafting efficiency.

While most studies use a single initiator in grafting reactions, here, two initiators were used to increase the grafting efficiency. In this unique strategy, the reaction temperature was increased to allow for an appropriate amount of the DBPH initiator to decompose, and then the temperature was lowered, and the BPO initiator was added. The cooling step in the reaction was similar to the quenching pretreatment used to swell the PP particles. After cooling, new active sites were generated, and the grafting polymerization continued, which in turn improved the grafting ratio and grafting efficiency.

Since both the initiator and grafted monomer used in this study were oil-soluble, xylene was chosen as the interfacial treatment agent. As can be seen from Figure 3C, the grafting ratio increased with the xylene dosage, and the maximum grafting rate was obtained using 3.5 wt% xylene relative to the PP. The increase in the grafting rate with the xylene concentration occurred because the added xylene swelled the amorphous regions of the PP, which promoted the diffusion of the initiator and monomer into the PP particles [30]. However, further increasing the xylene dosage above 3.5 wt% resulted in a slight decrease in the grafting rate because, at excessively high loading, the xylene coated the particle surface and prevented the diffusion of the monomers and initiators to the PP.

As can be seen from Figure 3D, the grafting ratio reached a maximum at 8 mL/g of dispersant to PP. A further increase in the amount of dispersant resulted in a slight decrease in the grafting rate. At low loadings, the dispersant could not ensure that the particles were well dispersed. During the reaction process, due to insufficient dispersion and adhesion, an independent suspension-grafting reaction system could scarcely be formed, and there were not enough sites for the reaction to occur. So, the grafting ratio was low at too-low loadings. At too high of a dispersant concentration, the number of individual particles, and therefore the reaction sites, was too high, and the relative concentration of the monomer to PP particles decreased. Therefore, the grafting ratio decreased at too high a dispersant concentration [31,32].

As can be seen from Figure 3E, both the grafting ratio and grafting efficiency increased with the initial monomer concentration. The highest grafting ratio was achieved when the total monomer dosage was 100% of the PP mass, while the highest grafting efficiency was achieved with a monomer dosage of 80% relative to the mass of PP. The likelihood of a monomer encountering a particle increased with the monomer concentration, which increased the grafting rate and efficiency. However, at too high concentration, the active sites on the main chain of PP that could participate in the grafting reaction were rapidly consumed due to a higher concentration of the monomer. On the other hand, the likelihood of a monomer encountering another monomer also increased, and therefore, the likelihood of side reactions also increased, and the grafting ratio and grafting efficiency decreased.

As can be seen from Figure 3F, as the AA-to-MAH mass ratio increased, the grafting ratio and grafting efficiency first increased and then slightly decreased. MAH struggles to self-polymerize because of its large-volume steric resistance, and AA self-polymerizes more easily. As the proportion of the AA monomer increased, the number of AA free radicals increased, as well as the likelihood of the AA free radicals coming into contact with the PP particles. The AA radical could also copolymerize with free MAH and then graft onto PP chain segments, which was also conducive to improving the grafting ratio and grafting efficiency. AA could also self-polymerize, and at too high an AA loading, the probability of an AA radical encountering another AA radical and a subsequent coupling termination reaction between AA radicals occurring also increased, resulting in an overall decrease in the grafting ratio and grafting efficiency. Moreover, the highly active AA monomers were more likely to react with the initiators at an excess AA loading, which reduced the probability of the initiators reacting with the PP and, thus, affected the grafting reaction.

### 3.3. Influence of Reaction Conditions on the Grafting Modification

**Figure 4:**
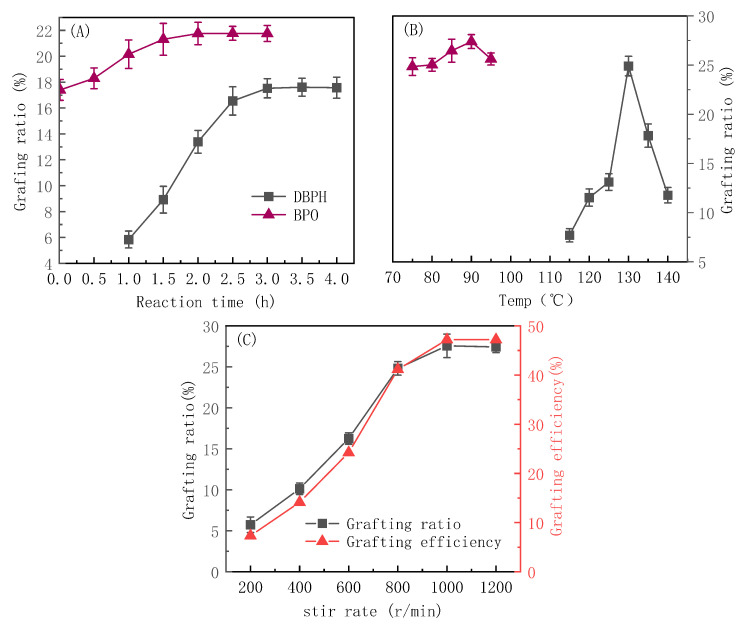
The effects of the reaction time (**A**), the reaction temperature (**B**), and the stir rate (**C**) on the grafting effect.

In addition to the reagent dosages, the reaction conditions also significantly impacted the grafting reaction. Therefore, this study also explored the effects of the reaction time, reaction temperature, and stir rate on the grafting reaction, and the results are shown in Figure 4.

As can be seen from Figure 4A, the grafting ratio using either the DBPH or BPO initiators increased rapidly and then more slowly with an increasing reaction time, and it plateaued around 3 h and 2 h, respectively. As the reaction time increased, the number of free radicals formed via decomposition of the initiators increased, and the grafting reaction proceeded quickly. In addition, the relative monomer concentration was higher, which further increased the grafting ratio. The grafting ratio plateaued at long times because, once all of the initiators had decomposed and the concentration of active free radicals and monomers decreased, the reaction rate plateaued.

As seen in Figure 4B, the optimum reaction temperatures using DBPH and BPO are 130 °C and 90 °C, respectively. The grafting ratio decreased to varying degrees with a further increase or decrease in temperature. The nonmonotonic trend with temperature was due to competing effects on the reaction efficiency. On one hand, increasing the temperature promoted the swelling and dispersion of the PP particles, which promoted the suspension grafting reaction. On the other hand, the reaction temperature also determined the decomposition rate of the initiator [33]. The decomposition of the initiator was accelerated at higher temperatures, and as a result, more free radicals were produced, and the grafting reaction was faster. However, at too high a temperature, the initiator decomposed too rapidly, and the free-radical-coupling-termination and monomer-homopolymerization side reactions were more likely. Therefore, the grafting ratio decreased.

As can be seen from Figure 4C, the grafting ratio and grafting efficiency both increased rapidly with the stir rate before plateauing at high speeds. Stirring promoted the dispersion of the PP particles, and it also increased the degree of mixing and ensured even heat distribution, all of which promoted the grafting reaction. However, at very high stir speeds, there were limited gains with a further increasing rate, and the grafting ratio and grafting efficiency plateaued.

Based on the results presented above, the following were selected as optimum reaction conditions: 3.5 wt% interfacial agent, 8 mg/L of dispersant, 80% monomer content, a 0.8 mass ratio of AA to MAH, a 1000 r/min stir rate for the reactor, 3.5 wt% initiator DBPH grafting at 130 °C for 3 h, and 1 wt% initiator BPO grafting at 90 °C for 2 h, as summarized in Table 1. These conditions resulted in the highest PP-g-AA-MAH grafting rate of 31.2%.

### 3.4. Characteristics of the PP-g-AA-MAH Fibers

#### 3.4.1. SEM Analysis of the Modified Fibers

**Figure 5:**
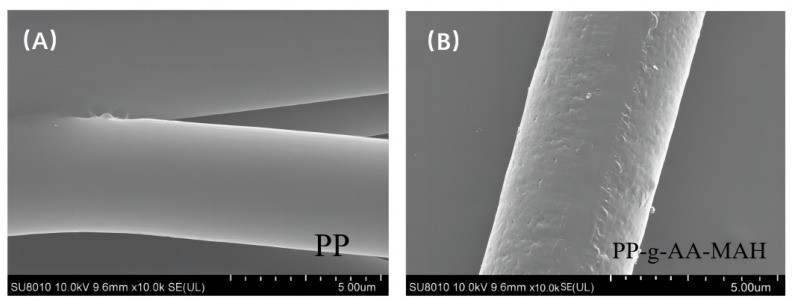
The micromorphology of the fibers before and after the suspension-grafting modification. (**A**) PP (**B**) PP-g-AA-MAH.

SEM images of the fiber morphologies before and after the modification treatment are shown in Figure 5. The fibers prepared with the unmodified PP had smooth surfaces (Figure 5A), while those prepared with the modified PP-g-AA-MAH polymers had rough and uneven surfaces (Figure 5B). The fiber diameter had clearly increased, and it was the evidence of the grafted polymer on the modified fiber surfaces. It is observed that PP was grafted with AA and MAH after suspension grafting polymerization.

#### 3.4.2. Fourier-Transform Infrared (FT-IR) Analysis of the Modified Fibers

**Figure 6:**
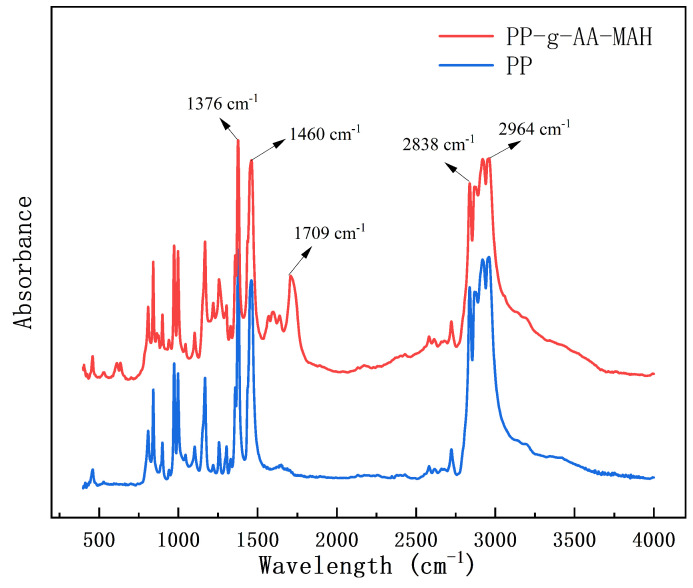
FT-IR spectra of the PP and PP-g-AA-MAH fibers.

Figure 6 shows the Fourier infrared spectra of the fibers prepared from either the neat PP or PP-g-AA-MAH. Note that the spectrum of PP-g-AA-MAH fibers contained the peaks characteristic of the PP fibers, as well as several new peaks. For example, the C-H symmetric and asymmetric -CH_3_ stretching vibration peaks at 2964 cm^−1^ and 2838 cm^−1^, respectively, the bending vibration absorption peaks from the CH_2_ groups at 1376 cm^−1^, and the absorption peaks characteristic of CH groups at 1460 cm^−1^ were present in both spectra. The new peak at approximately 1709 cm^−1^ in the spectrum of the PP-g-AA-MAH fibers was assigned as the stretching vibrations of the C=O in the carboxyl groups. The appearance of this peak confirmed that AA and MAH were successfully grafted to PP and that MAH was present as maleic acid.

#### 3.4.3. Nuclear Magnetic Resonance (^1^H-NMR) Analysis of the Modified Fibers

**Figure 7:**
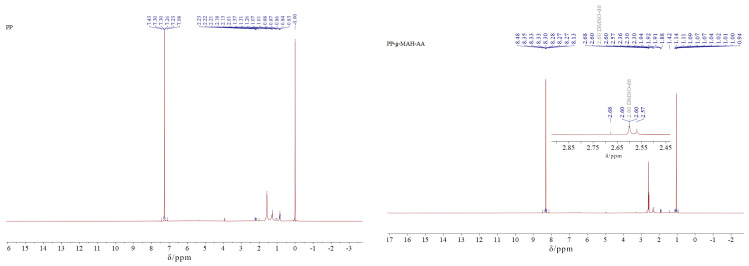
^1^H-NMR spectra of the PP and PP-g-AA-MAH fibers.

Figure 7 shows the ^1^H-NMR spectra of the PP and PP-g-AA-MAH fibers. In the PP-g-AA-MAH fibers, the carboxyl group was expected to be connected to -CH, and the chemical shift of -CH containing carboxyl substituents should be seen at approximately 2.6 ppm. As expected, a peak at approximately 2.6 ppm [34] was seen in the spectrum of the PP-g-AA-MAH fibers but not the PP fibers, which confirmed that the carboxyl groups were successfully grafted onto PP.

#### 3.4.4. Surface-Property Analysis of the Modified Fibers

**Figure 8:**
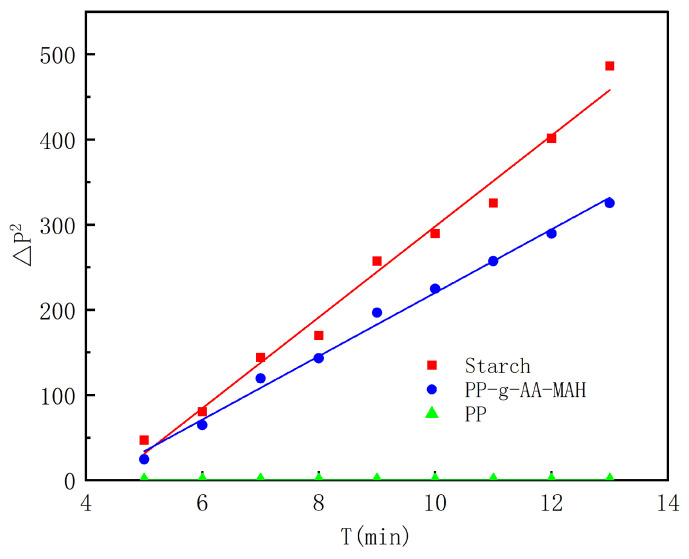
Measured ${\left(\Delta P\right)}^{2}-t$ curves for the PP and PP-g-AA-MAH fibers.

In this study, the capillary pressure method was used to test the hydrophilic properties of the product, first measuring the velocity of liquid penetration into the filling powder and then calculating the dynamic liquid contact angle using the Washburn [26] equation. The water contact angles were recorded using a micromanometer every 1 min. Curves of $\Delta P^{2}$ versus t for PP, PP-g-AA-MAH, and insoluble starch were recorded, as shown in Figure 8, where the contact angle of starch was defined as 0°. Because PP was hydrophobic, water did not penetrate into the capillary tube containing the PP powder, and no change in pressure was recorded. Therefore, the measured $\Delta P^{2}$ versus t was essentially constant. Meanwhile, both the starch and PP-g-AA-MAH fiber were hydrophilic, so water penetrated into the capillary, and the pressure changed over time. Based on these results, the water contact angle on the PP-g-AA-MAH fibers was determined to be 56.65°.

#### 3.4.5. Thermal Stability Analysis of the Modified Fibers

**Figure 9:**
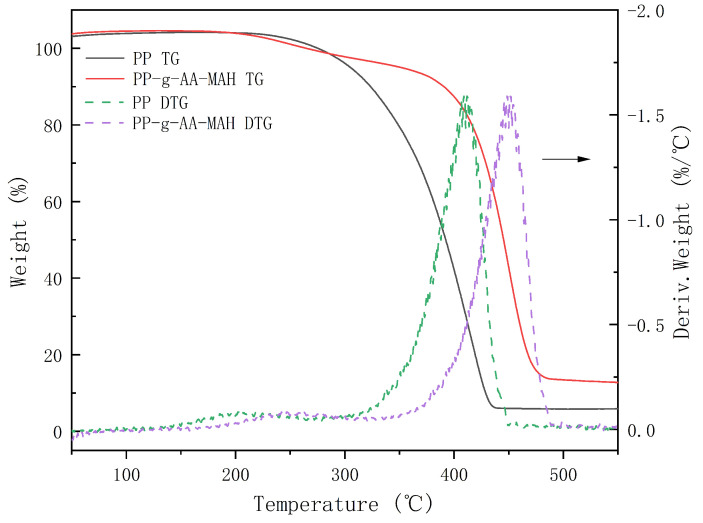
TG and DTG of PP and PP-g-AA-MAH fibers.

Figure 9 shows that the TG and DTG of PP and the graft product PP-g-AA-MAH fibers. With an increasing temperature, the grafted products lost weight before PP due to the exfoliation of AA and MAH copolymers on the product surface. Moreover, the maximum degradation temperature of PP was earlier than that of PP-g-AA-MAH, indicating that the grafting process had some cross-linking effect on the PP macromolecular chain. The maximum temperature of the melt-blown spinning process was 230 °C, while PP-g-AA-MAH had no obvious loss at 230 °C, indicating that the product had good thermal stability and would not produce decomposition during the melt-blown spinning process.

### 3.5. Adsorption and Regeneration of Indigo Dye Using the PP-g-AA-MAH Fibers

#### 3.5.1. Effect of pH on the Fiber Adsorption Capacity for Indigo

**Figure 10:**
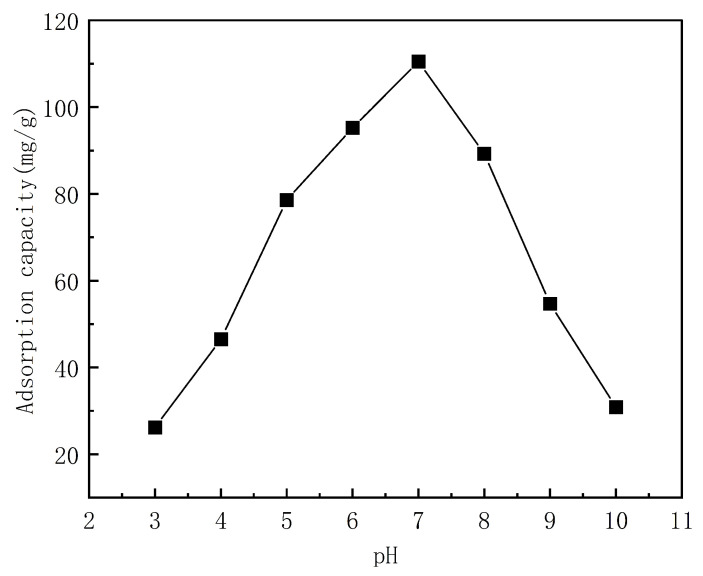
Effect of solution pH on the fiber adsorption capacity for indigo.

The pH of the solution can affect the surface charge and morphology of an adsorbent, and therefore, it is an important factor that influences the adsorption performance of a material. Therefore, the effects of the solution pH on the adsorption capacity of the PP-g-AA-MAH fibers at a constant adsorption temperature and adsorption time, as well as the initial concentration of the indigo solution of 25 °C, 2 h, and 200 mg/L, respectively, were studied. The results are shown in Figure 10.

As can be seen from Figure 10, the fiber adsorption capacity first increased and then decreased with an increasing pH. The highest adsorption capacity of 110.43 mg/g was measured for a solution pH of 7. The nonmonotonic trend with pH was because the charge state of the fibers and dyes changed with the pH. At a low-solution pH, the fibers were positively charged because of the high concentration of free H^+^ in the solution, and the indigo was only slightly soluble; therefore, the adsorption was not strong. The surface charge of the fibers gradually decreased with an increasing pH, and therefore, the number of -COO^−^ on the surface increased. These groups then interacted with the dye via electrostatic and hydrogen-bonding interactions, which increased the adsorption capacity of the fibers for the dye. At pH values greater than 7, the number of -COO^−^ groups on the fiber surface increased, and the indigo was either singly or doubly charged gradually under the action of insurance powder. The resulting electrostatic repulsion between the negatively charged fibers and dyes, therefore, decreased the adsorption capacity of the fibers at high pH values [26,35].

#### 3.5.2. Adsorption Kinetics

**Figure 11:**
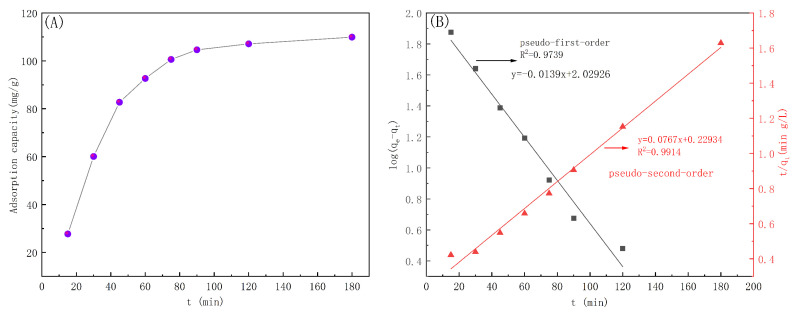
Adsorption capacity over time and (**A**) corresponding fits to pseudo-first-order and pseudo-second-order kinetic models (**B**).

At fixed conditions of an indigo solution concentration of 200 mg/L, a solution pH of 7, and a temperature of 25 °C, the adsorption kinetics were investigated. The measured adsorption capacities over time were fit with both pseudo-first-order and pseudo-second-order kinetic models [36], and the results are shown in Figure 11 and Table 2.

Figure 11 shows that the PP-g-AA-MAH fiber adsorption capacity for indigo first increased rapidly before plateauing at long times. At early time points, there were many active sites on the PP-g-AA-MAH fiber surface that could interact with the dye, and therefore, the adsorption rate was fast. The number of available binding sites on the fiber surfaces, as well as the concentration of free dye in the solution, decreased over time, and therefore, the adsorption rate gradually plateaued at long times. At long times, the active site on the surface of the fiber was saturated, and no additional dye could adsorb to the fibers.

The $R^{2}$ of the fit of the pseudo-first-order kinetic model to the experimental data was 0.9739, while that for the pseudo-second-order kinetic model was 0.9914, indicating that the pseudo-second-order model better described the data. The better agreement with the pseudo-second-order model suggests that the indigo dye adsorbed to the PP-g-AA-MAH fibers via chemisorption, and the number of available active sites controlled the adsorption rate.

#### 3.5.3. Adsorption Isotherms

**Figure 12:**
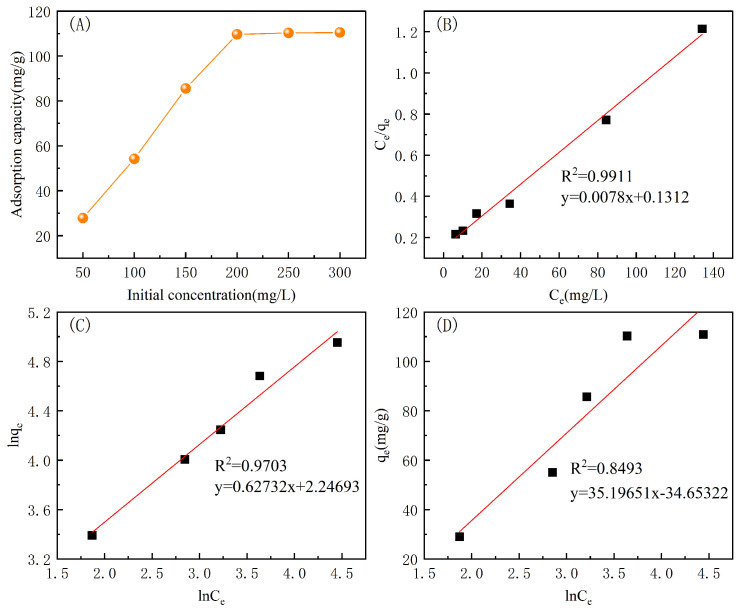
The effect of initial indigo concentration on the fiber adsorption capacity (**A**) and corresponding fits of the data to the Langmuir (**B**), Freundlich (**C**), and Temkin (**D**) models.

The effects of indigo concentration on the fiber adsorption capacity were investigated at 25 °C, pH = 7 with an adsorption time of 2 h. The measured isothermal adsorption isotherms were then fit with the Langmuir [37], Freundlich [38], and Temkin [39] adsorption models, and the results are shown in Figure 12 and Table 3.

As can be seen from the Figure 12, the adsorption capacity of the PP-g-AA-MAH fiber for indigo increased with the initial dye concentration in the solution. The adsorption capacity increased rapidly from 25.03 mg/g to 110.43 mg/g when the initial dye concentration increased from 50 mg/L and 200 mg/L. However, because there was a finite number of adsorption sites on the PP-g-AA-MAH fibers, the amount of adsorbed dye increased until the adsorption sites were saturated, at which point the amount did not increase further.

The linear correlation coefficient of the fit to the Langmuir isothermal adsorption model ($R^{2}$ = 0.9911) was closer to 1 than that of the Freundlich model ($R^{2}$ = 0.9703) and the Temkin model ($R^{2}$ = 0.8493). The best fit with the Langmuir isothermal adsorption model suggested that the PP-g-AA-MAH fibers had a uniform adsorption surface and all active sites had the same affinity for indigo. Moreover, the adsorption likely proceeded via monolayer adsorption. The maximum theoretical adsorption capacity from the fit to the data of 111.74 mg/g was also very close to the experimental value of 110.43 mg/g.

#### 3.5.4. Adsorption Performance of Regenerated PP-g-AA-MAH Fibers

**Figure 13:**
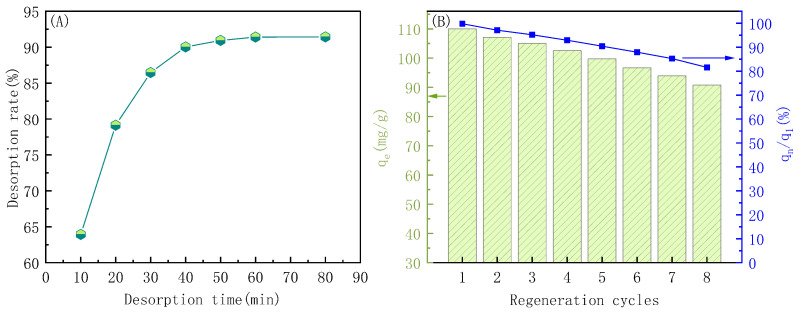
Effect of desorption time on the desorption rate of indigo (**A**); relationship between adsorption capacity and the regeneration cycle (**B**).

An ideal adsorbent has a high adsorption capacity for contaminants, but the contaminants can also be easily released (desorbed) from the material to reduce the material costs. Therefore, the desorption of indigo and regeneration of PP-g-AA-MAH fibers were investigated, and the results are shown in Figure 13.

As can be seen from Figure 13A, the amount of indigo released from the fibers increased over time before plateauing at 90.05% after 40 min. These results showed that the addition of a 0.5-mol/L HCl solution to the dye-saturated PP-g-AA-MAH fibers weakened the electrostatic and hydrogen-bonding interactions with the dye, and the dye was readily released back into the solution. Moreover, the PP-g-AA-MAH fibers were also highly acid-resistant and could be treated with a strongly acidic solution to remove the dye.

Figure 13B shows the adsorption capacity of PP-g-AA-MAH fiber for indigo after multiple adsorption/regeneration cycles. While the adsorption capacity decreased slightly after multiple use cycles, the adsorption capacity remained as high as 91.22 mg/g, or 81.7% of the original adsorption capacity, after eight use cycles. These results clearly show that the PP-g-AA-MAH fibers could be readily regenerated and reused to treat indigo wastewater.

#### 3.5.5. Comparison of Adsorption Capacity with Other Adsorbents

Comparisons between the maximum adsorption capacities ($q_{\max}$) of PP-g-AA-MAH fibers and other adsorbents for indigo reported in the literature are presented in Table 4. The results show that PP-g-AA-MAH fibers exhibit a reasonable capacity for indigo adsorption.

## 4. Conclusions

In the study presented here, modified PP-g-AA-MAH was prepared from PP melt-blow resin using a continuous suspension grafting strategy with AA and MAH functional monomers and a combination of DBPH and BPO initiators. Systematic experiments showed that the optimum modification conditions were as follows: 3.5 wt% xylene as an interfacial agent, 8 mg/L of water as a dispersant, 80% monomer content, a 0.8 mass ratio of AA to MAH, a 1000 r/min stir speed, 3.5 wt% initiator DBPH grafting at 130 °C for 3 h, and 1 wt% initiator BPO grafting at 90 °C for 2 h. The highest PP-g-AA-MAH grafting rate was 31.2%, as confirmed with FTIR and ^1^H NMR. Importantly, the modification increased the hydrophilicity of the fibers such that the water contact angle of the PP-g-AA-MAH fibers was 56.58°.

The prepared PP-g-AA-MAH fibers were then used to remove indigo from simulated wastewater from printing and dyeing processes. The solution pH value significantly influenced the PP-g-AA-MAH fiber adsorption capacity, and the highest adsorption capacity of 110.43 mg/g was measured at pH 7. The adsorption capacity increased rapidly with time before plateauing, and the indigo adsorption to the fibers was well described with a pseudo-second-order kinetic model and Langmuir isothermal adsorption model. Most notably, the dye could be readily desorbed from PP-g-AA-MAH fibers, and the adsorption capacity of the regenerated fibers reached 91.22 mg/g after 8 use cycles, which was 81.7% of the original adsorption capacity. Together, these results highlight that the PP-g-AA-MAH fibers prepared here, with excellent adsorption, desorption, regeneration, and reuse properties, effectively and efficiently removed indigo from wastewater.

## Figures and Tables

**Table 1:** Optimum process parameters for the suspension grafting reaction.

PP	Monomers		Xylene	DBPH			BPO			Water	RPM	Grafting Ratio
	**AA**	**MAH**		**Amount**	**Temp**	**Time**	**Amount**	**Temp**	**Time**			
200 g	71 g	89 g	700 mL	7 g	130 °C	3 h	2 g	90 °C	2 h	1.6 L	1000 r/min	31.2%

**Table 2:** Fit parameters for pseudo-first-order and pseudo-second-order kinetic models.

Kinetic Model	$q_{e}$ (mg/g)	K	$R^{2}$
Pseudo-first-order kinetic model	92.45	0.062	0.9739
Pseudo-second-order kinetic model	127.66	0.00078	0.9914

**Table 3:** Extracted parameters from fits to the Langmuir, Freundlich, and Temkin isotherm models.

Langmuir			Freundlich			Temkin		
$Q_{m}$ **/mg/g**	$K_{L}$	$R^{2}$	n	$K_{F}$	$R^{2}$	$B_{T}$	$K_{T}$	$R^{2}$
111.74	1.72	0.9911	2.0253	5.2069	0.9703	28.9843	0.5369	0.8493

**Table 4:** Adsorption capacity of various adsorbents for indigo.

Adsorbents	q_max_/mg·g−1	Reference
Chitosan-modified flamboyant pods (CMFP)	22.45	[40]
Coriander seeds	124	[41]
Nanocelluloses using acid hydrolysis and oxidizing agents	39.7	[42]
A composite hydrogel (DMCHA)	306.08	[43]
Zizyphus joazeiro Mart peel (ZJP)	50	[44]
Natural clay (NC)	57	[45]
CaO nanoparticles made from eggshells	260	[46]
Carbonized sugarcane bagasse	8.88	[47]
Spirulina platensis	91	[48]
PP-g-AA-MAH fibers	110.43	This work

## Data Availability

The original contributions presented in the study are included in the article, further inquiries can be directed to the corresponding author.

## References

[1] Chen Y., Zhang Y., Yuan Y., Li H., Dong K., Li X. (2019). Progress Research on Treatment Methods of Printing and Dyeing Wastewater. IOP Conf. Ser. Earth Environ. Sci..

[2] Yang Q., Ren S., Zhao Q., Lu R., Hang C., Chen Z., Zheng H. (2018). Selective separation of methyl orange from water using magnetic ZIF-67 composites. Chem. Eng. J..

[3] Ren Z., Yang X., Zhang W., Zhao Z. (2023). Preparation, characterization and performance of a novel magnetic Fe-Zn activated carbon for efficient removal of dyes from wastewater. J. Mol. Struct..

[4] Shukla A.K., Alam J., Rahaman M., Alrehaili A., Alhoshan M., Aldalbahi A. (2020). A Facile Approach for Elimination of Electroneutral/Anionic Organic Dyes from Water Using a Developed Carbon-Based Polymer Nanocomposite Membrane. Water Air Soil Pollut..

[5] Olivo-Alanís D., Atilano-Camino M.M., García-González A., Humberto-Álvarez L., García-Reyes R.B. (2021). Chlorophyll-sensitized phenolic resins for the photocatalytic degradation of methylene blue and synthetic blue wastewater. J. Sol.-Gel Sci. Technol..

[6] Chowdhury M.F., Khandaker S., Sarker F., Islam A., Rahman M.T., Awual M.R. (2020). Current treatment technologies and mechanisms for removal of indigo carmine dyes from wastewater: A review. J. Mol. Liq..

[7] Gao J.Y., Gao F.Z., Zhu F., Luo X.H., Jiang J., Feng L. (2019). Synergistic coagulation of bauxite residue-based polyaluminum ferric chloride for dyeing wastewater treatment. J. Cent. South Univ..

[8] Wang Z., Chu Y., Zhao G., Yin Z., Kuang T., Yan F., Zhang L., Zhang L. (2023). Study of surface wettability of mineral rock particles by an improved Washburn method. ACS Omega.

[9] Yang Q., Zhang W., Zhang H., Li Y., Li C. (2011). Wastewater treatment by alkali bacteria and dynamics of microbial communities in two bioreactors. Bioresour. Technol..

[10] Pan Z., Tao D., Ren M., Cheng L. (2023). A Combinational Optimization Method for Efficient Production of Indigo by the Recombinant Escherichia coli with Expression of Monooxygenase and Malate Dehydrogenase. Foods.

[11] Dhas P.G.T.N., Gulyas H., Otterpohl R. (2015). Impact of powdered activated carbon and anion exchange resin on photocatalytic treatment of textile wastewater. J. Environ. Prot..

[12] Hashemian S., Hidarian M. (2014). Synthesize and characterization of sawdust/MnFe_2_O_4_ nano composite for removal of indigo carmine from aqueous solutions. Orient. J. Chem..

[13] Qiu M., Huang C. (2015). Removal of dyes from aqueous solution by activated carbon from sewage sludge of the municipal wastewater treatment plant. Desalin. Water Treat..

[14] Peng S., Zhang D., Huang H., Jin Z., Peng X. (2018). Ionic polyacrylamide hydrogel improved by graphene oxide for efficient adsorption of methylene blue. Res. Chem. Intermed..

[15] Mostafa M.M.H., Nkudede E., Sulemana H., Zhang B., Zhu K., Hu S., Gblinwon R.T., Kosiba A.A., Manickam S. (2021). Treatment of simulated printing and dyeing wastewater using ozone microbubble. E3S Web Conf..

[16] Benhalima T., Sadi A., Dairi N., Ferfera-Harrar H. (2024). Multifunctional carboxymethyl cellulose-dextran sulfate/AgNPs@ zeolite hydrogel beads for basic red 46 and methylene blue dyes removal and water disinfection control. Sep. Purif. Technol..

[17] Qian J., Han X., Yang S., Kuang L., Hua D. (2018). A strategy for effective cesium adsorption from aqueous solution by polypentacyanoferrate-grafted polypropylene fabric under *γ*-ray irradiation. J. Taiwan Inst. Chem. Eng..

[18] Mahboubizadeh S., Hasanzadeh M., Panahi F., Arzaqi Z. (2021). A Review of Processing Methods and Modification of Reinforcements Used in Polypropylene Composites. J. Iran. Chem. Eng..

[19] Correa-Aguirre J.P., Luna-Vera F., Caicedo C., Vera-Mondragón B., Hidalgo-Salazar M.A. (2020). The Effects of Reprocessing and Fiber Treatments on the Properties of Polypropylene-Sugarcane Bagasse Biocomposites. Polymers.

[20] Mandal D.K., Bhunia H., Bajpai P.K., Chaudhari C.V., Dubey K.A., Varshney L. (2016). Radiation-induced grafting of acrylic acid onto polypropylene film and its biodegradability. Radiat. Phys. Chem..

[21] Abdel Ghaffar A., El-Arnaouty M., Aboulfotouh M.E., Taher N., Taha A.A. (2014). Radiation graft copolymerization of butyl methacrylate and acrylamide onto low density polyethylene and polypropylene films, and its application in wastewater treatment. Radiat. Eff. Defects Solids.

[22] Nosova N., Roiter Y., Samaryk V., Varvarenko S., Stetsyshyn Y., Minko S., Stamm M., Voronov S. (2004). Polypropylene surface peroxidation with heterofunctional polyperoxides. Macromol. Symp..

[23] Basuki B., Suyitno S., Kristiawan B. (2018). Absorbance and electrochemical properties of natural indigo dye. AIP Conference Proceedings.

[24] Li Z., Ma Y., Yang W. (2013). A facile, green, versatile protocol to prepare polypropylene-g-poly(methyl methacrylate) copolymer by water-solid phase suspension grafting polymerization using the surface of reactor granule technology polypropylene granules as reaction loci. J. Appl. Polym. Sci..

[25] Ji L., Shi B. (2015). A novel method for determining surface free energy of powders using Washburn’s equation without calculating capillary factor and contact angle. Powder Technol..

[26] Qing C., Bigui W., Yingdong H. (2009). Capillary pressure method for measuring lipophilic hydrophilic ratio of filter media. Chem. Eng. J..

[27] Lian Z., Xu Y., Zuo J., Qian H., Luo Z., Wei W. (2020). Preparation of PP-g-(AA-MAH) Fibers Using Suspension Grafting and Melt-Blown Spinning and its Adsorption for Aniline. Polymers.

[28] Bora D., Dutta H., Saha B., Reddy Y.A.K., Patel R., Verma S.K., Sellamuthu P.S., Sadiku R., Jayaramudu J. (2023). A review on modification of polypropylene carbonate (PPC) using different types of blends/composites and its advanced uses. Mater. Today Commun..

[29] Yang M., Sun L., Liu Q., Deng Y., Liu C., Li N., Xu J., Chen Y., Jian X. (2024). Ultra high density carbon fiber derived from isotactic polypropylene fiber without skin-core structure. Polym. Adv. Technol..

[30] George G., Joseph K., Saritha A., Nagarajan E.R. (2017). Influence of fiber content and chemical modifications on the transport properties of PP/jute commingled biocomposites. Polym. Compos..

[31] Jiao W., Liu W., Yang F., Jiang L., Jiao W., Wang R. (2017). Improving the interfacial property of carbon fiber/vinyl ester resin composite by grafting modification of sizing agent on carbon fiber surface. J. Mater. Sci..

[32] Liang F., Yuan H., Shao Q., Song W. (2018). Study of suspension grafting process of polypropylene. Des. Monomers Polym..

[33] Wang S., Muiruri J.K., Soo X.Y.D., Liu S., Thitsartarn W., Tan B.H., Suwardi A., Li Z., Zhu Q., Loh X.J. (2023). Bio-Polypropylene and Polypropylene-based Biocomposites: Solutions for a Sustainable Future. Chem. Asian J..

[34] Badertscher M., Bühlmann P., Pretsch E. (2009). Structure Determination of Organic Compounds: Tables of Spectral Data.

[35] Wang J., Sun C., Xia W., Cao Z., Sheng G., Xie X. (2022). Pd/BN catalysts for highly efficient hydrogenation of maleic anhydride to succinic anhydride. Appl. Catal. A Gen..

[36] Musah M., Azeh Y., Mathew J.T., Umar M.T., Abdulhamid Z., Muhammad A.I. (2022). Adsorption kinetics and isotherm models: A review. CaJoST.

[37] Yuan H., Shao Q., Liang F., Shi H., Song W. (2020). Mechanism of crosslinking in benzoyl peroxide-initiated functionalization of vinyltriethoxysilane onto polypropylene in the water medium. J. Appl. Polym. Sci..

[38] Zhao D., Wang L., Zhang R., Ma Y., Chen D., Zhao C., Yang W. (2018). Preparation of toughened polypropylene-g-poly (butyl acrylate-co-acrylated castor oil) by suspension grafting polymerization. Polym. Eng. Sci..

[39] Zhong J., Yang B., Feng Y., Chen Y., Wang L.G., You W.D., Ying G.G. (2021). Enhanced photo–Fenton removal efficiency with core-shell magnetic resin catalyst for textile dyeing wastewater treatment. Water.

[40] Okoya A.A., Diisu D.O., Olaiya O.O., Adegbaju O.S. (2020). Adsorption Efficiency of Flamboyant Pods for Indigo Dye Removal from Textile Industrial Wastewater. Environ. Pollut..

[41] Kadiri L., Ouass A., Hsissou R., Safi Z., Wazzan N., Essaadaoui Y., Lebkiri I., El Khattabi O., Housseine Rifi E., Lebkiri A. (2021). Adsorption properties of coriander seeds: Spectroscopic kinetic thermodynamic and computational approaches. J. Mol. Liq..

[42] Basta A.H., Lotfy V.F. (2022). Impact of pulping routes of rice straw on cellulose nanoarchitectonics and their behavior toward Indigo dye. Appl. Nanosci..

[43] Lv A., Lv X., Xu X., Chen Y., Zhang J., Shao Z.-B. (2024). Tailored multifunctional composite hydrogel based on chitosan and quaternary ammonium ionic liquids@montmorillonite with different chain lengths for effective removal of dyes and 4-nitrophenol. Sep. Purif. Technol..

[44] Ribeiro J. (2019). Study of the Ziziphus Joazeiro Peel for Indigo Blue Adsorption. Int. J. Adv. Res..

[45] Missaoui M., Tahri N., Daramola M.O., Duplay J., Schäfer G., Ben Amar R. (2019). Comparative investigation of indigo blue dye removal efficiency of activated carbon and natural clay in adsorption/ultrafiltration system. Desalin. Water Treat..

[46] Radhi I.K., Hussein M.A., Kadhim Z.N. (2019). Investigation of ni grosine, alizarin, indigo and acid fuchsin removal by modification of CaO derived from eggshell with AgI: Adsorption, kinetic and photocatalytic studies. Eur. J. Chem..

[47] Sanda O., Amoo K.I., Taiwo E.A. (2018). Studies on the adsorption of indigo dye from aqueous media onto carbonised sugarcane bagasse. Niger. J. Basic Appl. Sci..

[48] Robledo-Padilla F., Aquines O., Silva-Núñez A., Alemán-Nava G.S., Castillo-Zacarías C., Ramirez-Mendoza R.A., Zavala-Yoe R., Iqbal H.M.N., Parra-Saldívar R. (2020). Evaluation and Predictive Modeling of Removal Condition for Bioadsorption of Indigo Blue Dye by *Spirulina platensis*. Microorganisms.

